# A Case of Refractory Kawasaki Disease With Yersinia pseudotuberculosis Infection Successfully Treated With Cefotaxime Following Immunosuppressive Therapy

**DOI:** 10.7759/cureus.73866

**Published:** 2024-11-17

**Authors:** Koji Yokoyama, Masahiko Sakabe, Mitsukazu Mamada

**Affiliations:** 1 Department of Pediatrics, Japanese Red Cross Wakayama Medical Center, Wakayama, JPN

**Keywords:** immunosuppressive treatment, infliximab, kawasaki disease (kd), steroid use, yersinia pseudotuberuculosis infection

## Abstract

Kawasaki disease (KD) is a vasculitis mainly affecting children under five, with symptoms such as persistent fever, rash, red lips, strawberry tongue, conjunctivitis, and swollen hands and feet. Diagnosis is based on a fever lasting over five days plus at least four of these symptoms. Treatment includes intravenous immunoglobulin (IVIG) and aspirin to reduce complications, especially coronary artery issues.* Yersinia pseudotuberculosis (Y. ptsb.)* infection is a gram-negative bacterium. In KD patients, the most prevalent gastrointestinal symptoms were vomiting (28.9%), abdominal pain (17.4%), and diarrhea (16.9%). By contrast, diarrhea is observed in over 50% of patients with *Y. ptsb.* infection. *Y. pstb.* is a gram-negative bacterium reported to infect a wide variety of animal hosts and contact with these animals can serve as a potential clue in diagnosis. *Y. pstb.* infection can mimic KD with similar fever and rash symptoms, posing a diagnostic challenge. In practice, however, differentiation remains challenging. Differentiating between KD and *Y. ptsb.* is essential, especially in cases resistant to typical KD treatment. Distinguishing KD from *Y. pstb.* infection in clinical practice is crucial to prevent misdiagnosis, avoid unnecessary immunosuppression, and minimize delays in effective treatment. Misinterpreting *Y. pstb.* infection as KD may lead to inappropriate treatment strategies that fail to address the underlying infection, potentially resulting in adverse patient outcomes. Accurate and timely diagnosis is therefore essential to initiate appropriate therapeutic interventions. A 16-month-old boy presented with fever and diarrhea and was initially treated for infectious gastroenteritis, with elevated inflammatory markers noted (C-reactive protein (CRP) 4.47 mg/dL, white blood cell (WBC) 8,200/μL). As his condition progressed, he developed symptoms consistent with KD, including a rash and mucous membrane changes, and was treated with IVIG and aspirin. However, the fever persisted, and elevated inflammatory markers continued (CRP 3.93 mg/dL, WBC 9,700/μL), prompting additional immunosuppressive therapies for refractory KD. Ultrasound revealed gastrointestinal and lymph node abnormalities suggestive of vasculitis. Eventually, *Y. ptsb.* infection was confirmed through serology, and antibiotic treatment was reintroduced, leading to defervescence. This case highlights the challenge of distinguishing KD from *Y. ptsb.* infection because they can coexist, complicating treatment decisions. Rapid diagnostic methods for *Y. ptsb.*, specifically through loop-mediated isothermal amplification-polymerase chain reaction (LAMP-PCR) testing, are crucial to guide timely treatment, particularly given the risk of coronary artery complications associated with both conditions.

## Introduction

Kawasaki disease (KD) is one of the most prevalent vasculitic syndromes, but its cause remains unknown despite various theories involving environmental, infectious, and genetic factors [1]. *Yersinia pseudotuberculosis* (*Y. ptsb.*) is a Gram-negative bacterium of the genus *Yersinia* in the family Enterobacteriaceae. Although infections of *Y. ptsb. *occur less frequently than those of its relative *Yersinia enterocolitica (Y. ent.)*, they are commonly observed in infants [2]. *Y. ptsb.* infection is more common in children aged five to 15 and occurs more frequently in males than in females. *Y. ptsb.* transmitted primarily through contaminated food or water, with the most common sources being raw or undercooked pork, unpasteurized milk, and contaminated water. The infection is more prevalent in colder months and can proliferate at refrigeration temperatures [2,3]. *Y. ptsb.* infection presents with five primary clinical syndromes, namely, acute gastroenteritis, mesenteric lymphadenitis, erythema nodosum, septicemia, and reactive arthritis [3,4]. Notably, *Y. ptsb.* infection can manifest with clinical features that resemble KD, such as high fever, generalized rash, and lymphadenopathy, and other symptoms that overlap with the diagnostic criteria for KD [5]. There have been reports suggesting that the incidence of coronary artery lesions is higher in cases of KD associated with *Y. ptsb.* infection. This has led to the hypothesis that *Y. ptsb.* infection might be a predisposing or aggravating factor in the development of KD [6]. Immunomodulatory treatments, particularly intravenous immunoglobulin (IVIG), are administered to manage KD [7]. Antimicrobial therapies, including the use of antibiotics, are administered for *Y. ptsb.* infections [2]. We encountered a case of *Y. ptsb.* infection in which, despite administering immunomodulatory treatment for KD, the patient experienced persistent fever and gastrointestinal symptoms, ultimately necessitating antibiotic therapy. We report this case in which we were able to track the progression of symptoms using cytokine analysis and abdominal ultrasound. Furthermore, we discuss the necessity of rapid testing for *Y. pstb.* infection, specifically through loop-mediated isothermal amplification-polymerase chain reaction (LAMP-PCR) testing.

## Case presentation

A 16-month-old male child, weighing 10.6 kg, presented to our hospital with a two-day history of fever and watery diarrhea. He had a prior history of vaccinations, including administration of the Bacillus Calmette-Guérin (BCG) vaccine. He did not keep pets and did not use well water. His blood pressure measured 110/64 mmHg, with a pulse rate of 180 beats per minute, a respiratory rate of 48 breaths per minute, a tympanic temperature of 40.6°C, and an oxygen saturation of 100% on ambient air. Laboratory results revealed a white blood cell (WBC) count of 8,200/μL, with an absolute neutrophil count of 5,723/μL and an absolute lymphocyte count of 1,681/μL. C-reactive protein (CRP) was notably elevated at 4.47 mg/dL, aspartate aminotransferase (AST) was mildly elevated at 63 U/L, and albumin was decreased to 4.0 g/dL. In addition, inflammatory cytokine levels were markedly elevated (Table 1).

**Table 1:** Laboratory parameters of the patient with reference range BT: body temperature, CRP: C-reactive protein, AUS: abdominal ultrasound examination, IVIG: intravenous high-dose immunoglobulin, IFX: infliximab, CTX: cefotaxime, ASA: aspirin, CsA: cyclosporin A, PSL: prednisolone, UTI: ulinastatin, KD: Kawasaki disease, IL: interleukin, CXCL-9: C-X-C motif chemokine ligand, sTNFRⅡ: soluble tumor necrosis factor receptor II

Laboratory investigations	Values	Biological reference range
WBC	8,200	4,500〜13,500 /μL
Neutrophil	5,723	1,500〜8,000 /μL
Lymphocyte	1,681	1,500〜8,000/μL
CRP	4.47	0.0〜0.3 mg/dL
AST	63	30～50 IU/L
Alb	4.0	4.5〜6.0 g/dL
IL-6	92	<3 pg/mL
IL-18	408	< 500 pg/mL
CXCL-9	2,890	<31-83 pg/mL
sTNFR II	11,373	<829-2,262 pg/mL

**Figure 1:**
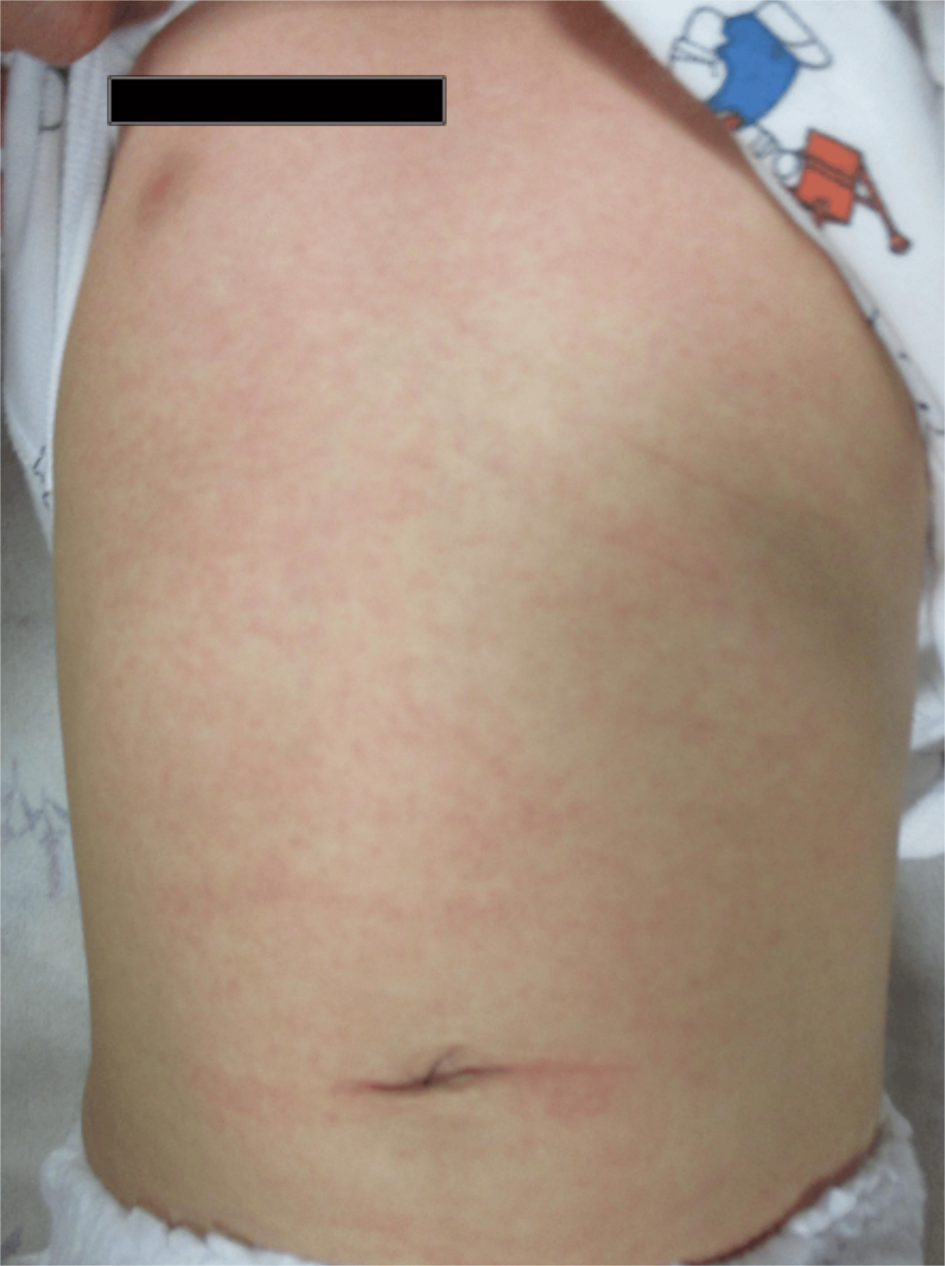
Non-pruritic infiltrative erythema was observed on the patient's trunk following admission.

After hospitalization, infectious gastroenteritis was suspected, and antibiotic therapy was initiated from the second day of illness. No rash, changes in the extremities, conjunctival injection, or oral changes were observed. He persisted with a fever, and further symptoms developed, including a maculopapular rash, erythematous cracked lips, a strawberry-like tongue, erythema of the oral mucosa, swollen and erythematous extremities, conjunctival injection, and bilateral cervical lymphadenopathy (Figure 1).

**Figure 2:**
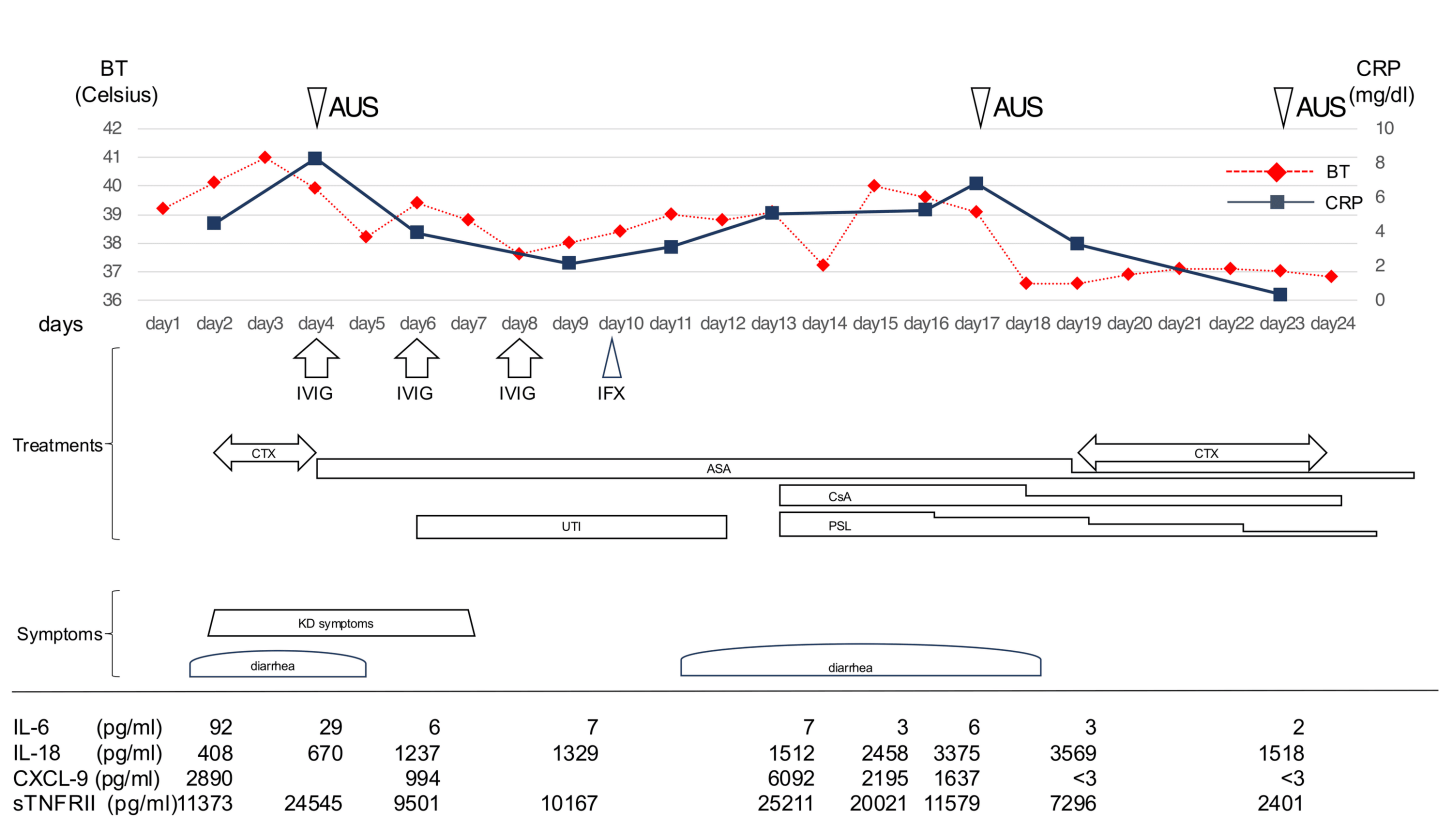
Clinical course of the acute phase of KD and Y. ptsb. infection, including inflammatory markers and treatments. BT: body temperature, CRP: C-reactive protein, AUS: abdominal ultrasound examination, IVIG: intravenous high-dose immunoglobulin, IFX: infliximab, CTX: cefotaxime, ASA: aspirin, CsA: cyclosporin A, PSL: prednisolone, UTI: ulinastatin, KD: Kawasaki disease, IL: interleukin, CXCL-9: C-X-C motif chemokine ligand, sTNFRⅡ: soluble tumor necrosis factor receptor II

The patient was diagnosed with KD on the fourth day of persistent fever and received treatment with an intravenous infusion of immunoglobulin at a dose of 2 g/kg alongside aspirin at 50 mg/kg. Following this intervention, antibiotic therapy was discontinued. On the sixth day, he was still febrile, his skin rash extended, and his inflammatory markers remained elevated (CRP 3.93 mg/dL, WBC 9,700/μL); hence, IVIG and uristatin (10,000 units/kg/day) were administered a second time. His symptoms, except fever, subsided. On the eighth, 10th, and 13th days, IVIG and infliximab (5 mg/kg), prednisone (PSL 1.5 mg/kg), and cyclosporin A (CsA, 5 mg/kg) were administered, respectively. The patient's fever persisted, and around the 10th day of illness, the diarrhea, which had previously improved, worsened again (Figure 2).

**Figure 3:**
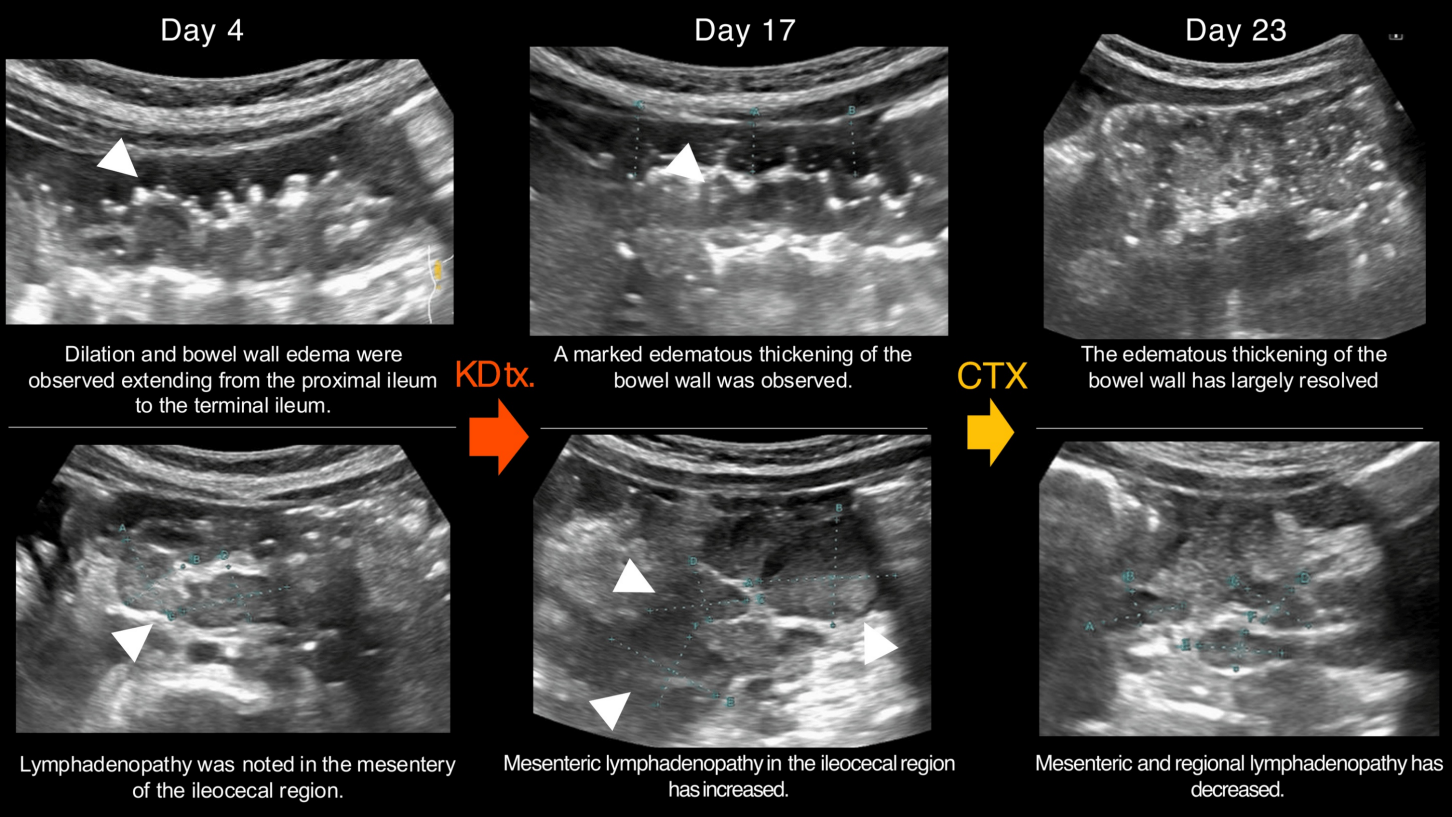
Progression of abdominal ultrasonography following the patient's admission. The upper panel illustrates the intestinal tract surrounding the terminal ileocecal region, while the lower panel depicts the mesenteric lymph nodes of the ileocecal region. KD: Kawasaki disease, CTX: cefotaxime On day 4 of the illness, enlarged mesenteric lymph nodes were observed. On day 17, edema of the intestinal wall was noted in the upper section. On the same day, further enlargement of the lymph nodes compared to day 4 was observed in the lower section. By day 23, improvement in intestinal wall edema was seen in the upper section. On the same day, marked improvement in lymph node enlargement was noted in the lower section.

We conducted a time-series examination of the patient's abdominal ultrasound. On the seventh day of illness, we observed dilation and edema of the intestinal wall in the ileum and colon. In addition, the mesentery of the ileocecal region was edematous and thickened, with multiple swollen lymph nodes, suggestive of systemic vasculitis, including KD. By the 17th day, there was marked hyperemia of the colonic wall, and the lymph node swelling in the ileocecal mesentery had further intensified compared with the previous examination. The edematous thickening of the gastrointestinal wall also became more pronounced, suggestive of bacterial enteritis (Figure 3).

Based on these findings, cefotaxime was re-administered. The patient defervesced the next day and was afebrile late on with the improvement of inflammatory markers. Although stool and blood cultures did not detect any significant pathogens, analysis of *Yersinia* serum agglutinin titers revealed a result below detectable sensitivity on the second day of illness and a 640-fold increase on the 17th day. Based on these findings, the patient was diagnosed with *Y. ptsb.* infection (serotype 4b) [8]. Interleukin-6 (IL-6) and interferon-gamma (IFN-γ) are cytokines implicated in the immunopathogenesis of KD, with IL-6 playing a key role in its progression, while the necessity of IFN-γ for disease induction remains unclear [9,10]. C-X-C motif chemokine ligand 9 (CXCL9) is an IFN-γ-inducible chemokine: measuring levels of CXCL9 rather than IFN-γ is advantageous because IFN-γ quickly binds to target structures or is neutralized by soluble receptors [11]. Cytokine analysis in this case indicates that while the KD itself was alleviated through immunomodulatory and immunosuppressive therapy, inflammation persisted due to other factors and was ultimately resolved completely with the administration of antibiotics. His electrocardiogram demonstrated robust cardiac contractility and increased perivascular echogenicity of the coronary arteries, without coronary artery dilatation or pericardial effusion for approximately the first month, but no further abnormalities were subsequently noted during outpatient follow-up for five years.

## Discussion

Most cases of *Yersinia* infections are self-limiting and do not require antibiotics, but appropriate antibiotic therapy is critical in severe cases [2]. According to international guidelines and the recommendations of the Japan Pediatric Society, the use of antibiotics is generally not recommended for the treatment of KD [7,12]. Furthermore, it was reported that the likelihood of IVIG resistance rises with a higher CRP level and utilization of multiple intravenous antibiotics [13]. *Y. pstb.* infection and KD are often challenging to distinguish and can coexist in some cases, such as the one presented here [14]. In KD patients, the most prevalent gastrointestinal symptoms were vomiting (28.9%), abdominal pain (17.4%), and diarrhea (16.9%) [15]. By contrast, diarrhea is observed in over 50% of patients with *Y. ptsb.* infection [2]. *Y. pstb. *is a gram-negative bacterium reported to infect a wide variety of animal hosts and contact with these animals can serve as a potential clue in diagnosis. In practice, however, differentiation remains challenging [16]. Regarding the relationship between KD and *Y. pstb.* infection, approximately 10% of 452 cases diagnosed with KD were serologically confirmed to have* Y. pstb.* infection. On the other hand, of 462 cases of *Y. pstb.* infection, 59 (13%) met the diagnostic criteria for KD, with 13 of these cases being definitively diagnosed. Some reports suggest that the incidence of coronary artery lesions is higher in cases of KD associated with *Y. pstb.* infection. *Y. pstb.* infection may be a predisposing or exacerbating factor for KD; therefore, rapid and appropriate treatment for affected patients is essential [14]. In cases of *Y. pstb.* infection that meet the diagnostic criteria for KD, consideration of aggressive KD treatment is recommended [14].

Tumor necrosis factor (TNF) is essential in defending against infections caused by intracellular pathogens. However, the widespread clinical application of anti-TNF therapy for managing autoinflammatory conditions has been linked to a heightened risk of severe infections. Although *Y. pstb.* is predominantly an extracellular pathogen, TNF is essential for host defense against it [17]. Therefore, immunosuppressive therapies such as anti-TNF blockers, PSL, and CsA may exacerbate infections, including those caused by *Yersinia* [18,19]. Numerous pathogens, besides *Yersinia*, have been reported in association with KD [20]. In cases where infections are implicated in KD, the primary focus remains on the treatment of KD itself [21]. Among the few reports of pathogens causing complications related to KD treatment are cases associated with *Y. ent.* infection, which has shown resistance to immunosuppressive therapy but ultimately resolved following antibacterial treatment [16]. This report, along with ours, represents the only documented cases in which IVIG and immunosuppressive therapy were administered for refractory KD associated with *Yersinia* infection. However, with the potential for immunosuppressive therapies to be more actively implemented as adjunctive treatments to IVIG, it is anticipated that infections as complications may increasingly emerge as a concern.

The gold standard for diagnosing *Yersinia* infection is culture; however, this is challenging in practice. Culturing *Yersinia* can be difficult because the bacteria are small, present in low numbers, and can be hard to distinguish from other nonpathogenic bacteria [22]. Regarding the diagnosis of *Y. pstb.* infection, agglutination titers increase within one to four weeks after onset and peak around day 40. A fourfold rise of the titer in paired sera or a single titer of 1:160 or higher is considered diagnostic [8]. At our institution, 10 cases of *Y. pstb.* infection have been diagnosed despite significant challenges in culturing this bacterium, with the diagnostic process relying on agglutination antibody titer measurements conducted at an external facility, which requires approximately 30.9 ± 13.0 days. Timely anti-inflammatory treatment is crucial during the acute phase of KD, and it is not feasible to wait 30 days or more for a definitive diagnosis. Ideally, a rapid method to analyze *Y. pstb.*, such as LAMP-PCR analysis or establishment of a stable *Y. pstb. *culture method, should be readily implementable. Physicians should consider the possibility of *Y. pstb.* infection in cases of KD accompanied by diarrhea and assess the need for antibiotic therapy.

## Conclusions

We encountered a case of KD complicated by *Y. pstb.* infection. While immunosuppressive therapies have become increasingly prevalent in the management of refractory KD, cases complicated by *Y. pstb.* infection present an elevated risk of persistent inflammation and coronary artery complications. This case highlights the critical importance of rapid and accurate diagnostic methods to identify *Y. pstb.* in KD cases unresponsive to conventional therapy. When KD does not improve with standard treatments, *Y. pstb.* should be considered in the differential diagnosis to minimize the risks associated with delayed or inappropriate treatment. In addition to immunosuppressive therapy, timely and appropriate use of antibiotics may be essential to address the underlying infection and avert further complications. Such an approach underscores the importance of precise diagnosis and intervention to improve patient outcomes in complex KD cases.
